# Electric field engineering and modulation of CuBr: a potential material for optoelectronic device applications

**DOI:** 10.1039/d3ra00157a

**Published:** 2023-03-07

**Authors:** Suneela Arif

**Affiliations:** a Department of Physics and Astronomy, Hazara University, Garden Campus (Main Campus) Mansehra Pakistan suneela@hu.edu.pk +092 310-050-7841

## Abstract

I–VII semiconductors, well-known for their strong luminescence in the visible region of the spectrum, have become promising for solid-state optoelectronics because inefficient light emission may be engineered/tailored by manipulating electronic bandgaps. Herein, we conclusively reveal electric-field-induced controlled engineering/modulation of structural, electronic and optical properties of CuBr *via* plane-wave basis set and pseudopotentials (pp) using generalized gradient approximation (GGA). We observed that the electric field (*E*) on CuBr causes enhancement (0.58 at 0.0 V Å^−1^, 1.58 at 0.05 V Å^−1^, 1.27 at −0.05 V Å^−1^, to 1.63 at 0.1 V Å^−1^ and −0.1 V Å^−1^, 280% increase) and triggers modulation (0.78 at 0.5 V Å^−1^) in the electronic bandgap, leading to a shift in behavior from semiconduction to conduction. Partial density of states (PDOS), charge density and electron localization function (ELF) reveal that electric field (*E*) causes a major shift and leads to Cu-1d, Br-2p, Cu-2s, Cu-3p, and Br-1s orbital contributions in valence and Cu-3p, Cu-2s and Br-2p, Cu-1d and Br-1s conduction bands. We observe the control/shift in chemical reactivity and electronic stability by tuning/tailoring the energy gap between the HOMO and LUMO states, such as an increase in the electric field from 0.0 V Å^−1^ → 0.05 V Å^−1^ → 0.1 V Å^−1^ causes an increase in energy gap (0.78 eV, 0.93 and 0.96 eV), leading to electronic stability and less chemical reactivity and *vice versa* for further increase in the electric field. Optical reflectivity, refractive index, extinction coefficient, and real and imaginary parts of dielectric and dielectric constants under the applied electric field confirm the controlled optoelectronic modulation. This study offers valuable insights into the fascinating photophysical properties of CuBr *via* an applied electric field and provides prospects for broad-ranging applications.

## Abstract 2

I–VII semiconductors are promising candidates for the solid-state optoelectronics may be engineered/tailored by manipulating electronic bandgaps.

## Graphical abstract

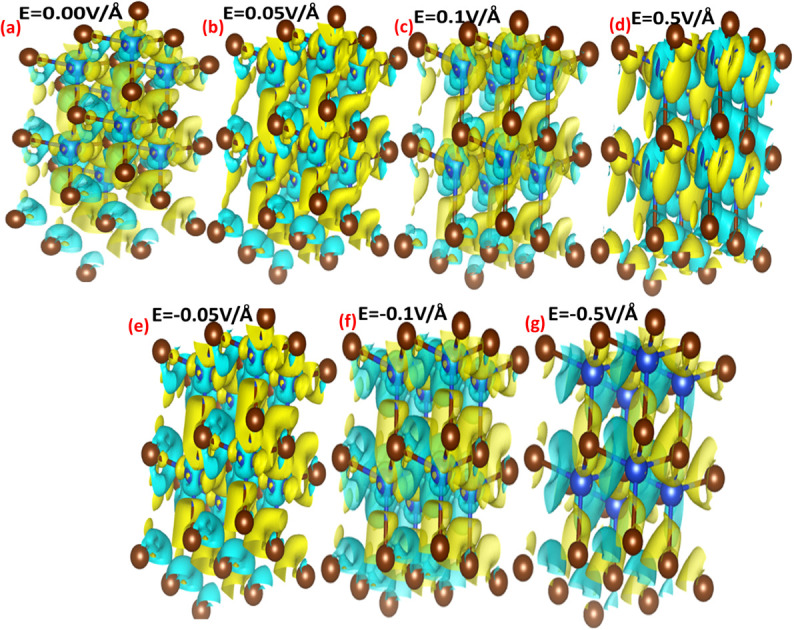

## Introduction

Wide band gap semiconductors renowned for strong luminescence in the visible region of the spectrum produce high concentrations of excessive charge carriers and have become prospective materials for optoelectronic applications.^1–3^ The majority of our daily lighting systems are based on InGaN optical emitters,^4,5^ while, ZnO,^6^ ZnS,^7^ ZnTe^8^ and ZnSe^9^ frequently used in flat panels and lasing applications suffer serious challenges. For example, the larger lattice mismatch with substrates, such as sapphire, Si, or SiC, direct to the intrinsic structural defects or distortions, such as misfit dislocations,^10^ stress in deposited films,^11^ in-phase (IPB) and out-of-phase grain boundaries (OPBs),^12^ and secondary impurity phases^13^ to the parent phases causes the hindrance in the light emitting efficiency of the devices. Additionally, excessive and continuous in use in household and luminescent optoelectronic devices causes scarcity, necessitating alternative novel materials with better or closer properties to in-based systems.^14,15^

I–VII Cu halides (CuX; X = Cl, Br, I, *etc.*) are well thought-out potential materials to replace In-based optoelectronic systems because of their structural diversity, direct band gap (3.0–3.5 eV at 300 K), transparency throughout the visible region (above 420 nm), large binding, smaller bulk modulus, large ionicity, diamagnetic behavior, non-linear optics, large excitonic binding (around 100 to 110 meV) energies (in the UV/visible region), negative spin orbit and rich excitonic photophysical/chemical optoelectronic properties (such as solid state lightening).^16–18^ These compounds are tetrahedral-coordinated compound semiconductors that crystallize into the zinc blend lattice (space group *F̄*43*m*), where Cu atoms are located at (0, 0, 0) and halide (F, Cl, Br, and I) atoms are located at (1/4, 1/4, 1/4) positions transformed from zinc blend (B_3_) to several intermediate low symmetry phases and rock salt (B_1_) structure under application of higher pressure (usually in the region of the 10 GPa).^19^ The filled d^10^-shell in addition to the s^2^p^6^ rare-gas valence shell originate valence band results in a deep core level with almost no dispersion apart from the spin–orbit splitting, which is part of the uppermost valence band, resulting in significant optoelectronic properties, as revealed recently that CuCl deposited on the Si substrate is a wideband gap (WBG) material compatible with the photoluminescence industry.^20,21^ Koch *et al.*^22^ briefly outlined the UV/vis emission spectral for copper-based halides and demonstrated that CuBr extends the probable range of blue hues in the recognized emitter wavelength range and have excellent lattice match with a substrate such as Si, resulting in fewer structural defects in the deposited CuBr films *via* high vacuum^23–25^ or chemical solution-based deposition (CSD) process^26–28^ and yield efficient room-temperature free-excitonic emission. These materials have been widely produced, but no thorough theoretical investigations have been conducted on intrinsic and extrinsic electric field modulation and controlled engineered optoelectronic, mechanical, dielectric, elastic and photocatalytic properties, which may massively affect the emission, and conduction can potentially be useful in the controlled miniaturized optoelectronic industry that requires thorough and comprehensive investigations.

Herein, we selected the CuBr compound, which is the first ever thorough report of the electric field modulation and engineering of the bandgap of CuBr with zinc blende cubic (*a* = 5.69 Å, *b* = 5.69 Å and *c* = 5.69 Å) sphalerite structure exhibiting *F̄*43*m* space group (where the Cu^1+^ is bonded to four equivalent Br^1−^ atoms to form corner-sharing CuBr_4_ tetrahedra; the Cu–Br bond lengths are 2.47 Å, and the Br^1−^ is bonded to four equivalent Cu^1+^ atoms to form corner-sharing BrCu_4_ tetrahedra) halides *via* plane-wave basis set and pseudopotentials (pp) using generalized gradient approximation (GGA)^32–34^ in QUANTUM ESPRESSO.^35–37^ Despite wide theoretical studies on semiconductors, very few studies are available on Cu-based halides to date. We first ever thoroughly report the external electric field modulation and engineering of the energy band gap and its effect on the structural, electronic and optical properties of CuBr, which may lead to potentially controlled novel miniaturized future optoelectronic and photoluminescence technology.

### Computational methods details

In this study, we report the external electric field (0.0 V Å^−1^, 0.05 V Å^−1^, 0.1 V Å^−1^, 0.5 V Å^−1^, 0.7 V Å^−1^, −0.05 V Å^−1^, −0.1 V Å^−1^, −0.5 V Å^−1^, −0.7 V Å^−1^) controlled modulation and engineering of the structure (electron localization function (ELF) and wave function) and electronic (bandgap, total density of states (TDOS) and partial density of states (PDOS)) and optical (refractive indices *n*(*ω*), optical reflectivity *R*(*ω*), extinction coefficient *k*(*ω*), dielectric constant *ε*(*ω*), real *ε*_1_(*ω*) and imaginary dielectric *ε*_2_(*ω*)) properties of Cu-based CuBr halides using generalized gradient approximation (GGA)^29–34^ within the framework of density functional theory (DFT) using Quantum Espresso core Plane-Wave basis set^35–37^ and pseudopotentials (pp).^38,39^ The exchange correlation energy functional is determined using the Perdew–Burke–Ernerhof (PBE) functional^40^ with generalized gradient approximation (GGA). The energy cutoff is set to 40 eV for plane wave expansion, and the first Brillouin zone is represented by a 6 × 6 × 6 *k*-points grid.

## Results and discussions

**Fig. 1:**
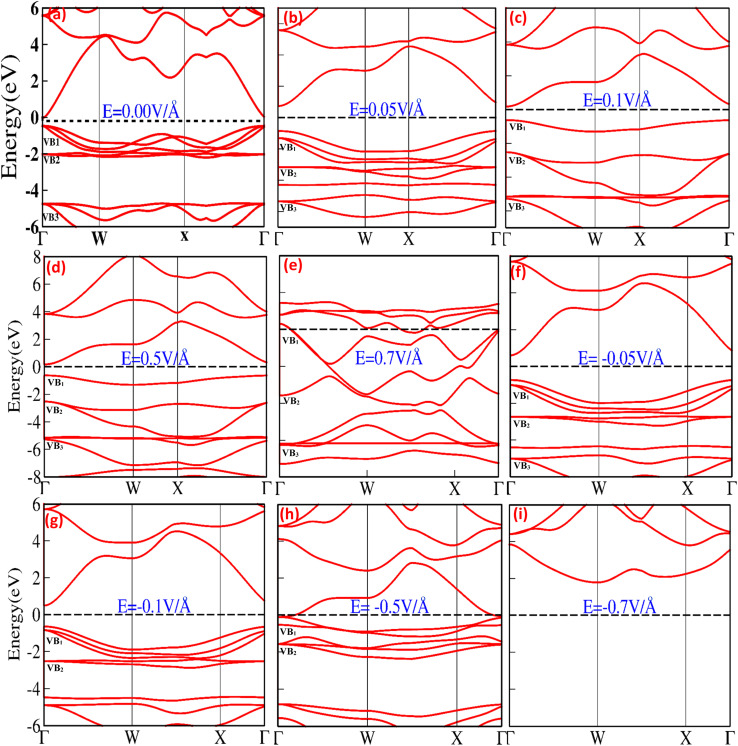
(a–i) Electric-field-induced modulation of electronic band structure of CuBr at (a) 0.00 V Å^−1^, (b) 0.05 V Å^−1^, (c) 0.1 V Å^−1^, (d) 0.5 V Å^−1^, (e) 0.70 V Å^−1^, (f) −0.05 V Å^−1^, (g) −0.1 V Å^−1^, (h) −0.5 V Å^−1^ and (i) −0.0 V Å^−1^ using the GGA scheme.

The electronic band structure and density of states (DOS) provide information about the electronic properties of materials that may be modulated, controlled, and engineered upon the application of an external electric field, offering a window of opportunity for potential utilization in micro/nanoelectronic technology. Fig. 1(a–i) shows the bandgap modulation and engineering for CuBr halides with (0.05 V Å^−1^, 0.1 V Å^−1^, 0.5 V Å^−1^, 0.7 V Å^−1^, −0.05 V Å^−1^, −0.1 V Å^−1^, −0.5 V Å^−1^ and −0.7 V Å^−1^) and without the application of an external electric field (0.0 V Å^−1^) using GGA approximation. In the absence of an external applied electric field (*E*) (Fig. 1(a)), the top of the valence band maximum (VBM) and the bottom of the conduction band minimum (CBM) are not overlapping located and diverge at the *Γ*–*Γ* symmetry point of the Brillouin zone (BZ) responsible for the direct band gap, *E*_g_, (0.58 eV at 0.0 V Å^−1^), validating semiconducting behavior that is consistent with previously reported results.^17,19,20,41^ The direct band gap at 0.0 V Å^−1^ appears owing to the main interaction between Cu metal d-states and Br p-states, which pull down valence bands near the Fermi level (*E*_F_). It is clearly observed that the highest valence band (VB_1_ and VB_2_) states have d-state electron contributions, whereas the p-state electrons mainly contribute to the lowest (VB_3_) bands. The band structures below the critical external field (0.7 V Å^−1^ and −0.5 V Å^−1^) comprise the lower part, VB_1_, and flat part VB_2_, appearing because of the hybridization of 3d Cu_t2g_^+^ and 3p Br^−^ orbital (VB_2_) and 3p_eg_ of 3d Cu^+^ orbital (VB_2_). The lower lying VB_3_ band is mostly derived from the 3p (Br^−^) orbital, whereas the s (Br^−^) orbital coins the deeper VB_4_ band entirely. The shapes of the lower VB_3_ and VB_4_ bands are the same for the zinc blende materials, in which the d bands are far away from the valence band region.

Fig. 1(b–i) depicts that the application of an external electric field (V Å^−1^) causes modulation and tuning in the valence and conduction band, resulting in a shift and widening of the direct bandgap at *Γ* symmetry point below the critical electric field (0.05 V Å^−1^*E*_g_ = 1.58, −0.05 V Å^−1^*E*_g_ = 1.27 and 0.1 V Å^−1^, *E*_g_ = 1.63). The bandgap starts to shrink at 0.5 V Å^−1^ and −0.1 V Å^−1^ shifts from 1.63 to 0.78 and 1.27 to 1.13; above the critical field (0.7 V Å^−1^ and −0.5 V Å^−1^), conduction band crosses the Fermi energy level and overlaps valence band, resulting in a metallic response. The detailed computed values of the energy bandgap obtained as a function with and without an applied external electric field (above and below the critical electric field) for the CuBr system are listed in Table 1, confirming that CuBr responds sensitively and monotonously to the external applied electric field (V Å^−1^).

**Table 1:** Detailed values of the electric-field-induced electronic bandgap and engineering and modulating of the highest occupied molecular orbitals (HOMO) or bonding orbitals and lowest un-occupied molecular orbitals (LUMO) or anti-bonding orbital energy wave function properties of CuBr

S. no.	*E* (V Å^−1^)	*E*_g_ (eV)	*E*_CBM_ (eV)	*E*_VBM_ (eV)	*E*_HOMO_ (eV)	*E*_LUMO_ (eV)	Δ*E* (_LUMO–HOMO_)
1	0.00	0.58	0.11	0.47	6.4	7.18	0.78
2	0.05	1.58	0.75	0.83	5.1460	6.0835	0.937
3	0.10	1.63	0.75	0.88	4.5537	5.5148	0.961
4	0.50	0.78	0.22	0.56	−1.2446	−0.6009	−0.643
5	0.70	0.00	0.00	0.00	−4.7467	−4.0466	−0.700
6	−0.05	1.27	0.59	0.68	6.2595	6.9795	0.720
7	−0.10	1.13	0.50	0.63	0.6774	7.2961	0.517
8	−0.50	0.00	0.00	0.00	—	—	—
9	−0.70	0.00	0.00	0.00	—	—	—

The bandgap expanded and tuned from 0.58 (at 0.0 eV) to 1.63 (at 0.1 V Å^−1^) *via* applied external electric field (V Å^−1^) as stimuli provide a window of opportunity to develop, control, engineer and modulate future optoelectronic devices, possibly causing strong luminescence in the visible region of the spectrum because of the large bandgap producing a high concentration of excessive charge carriers. No previous theoretical and experimental studies have been conducted on electric field engineering and modulation of CuBr, and we believe this study will be used as a reference and has the potential for further investigation of the engineering, controlling and modulating of modern optoelectronic devices.

**Fig. 2:**
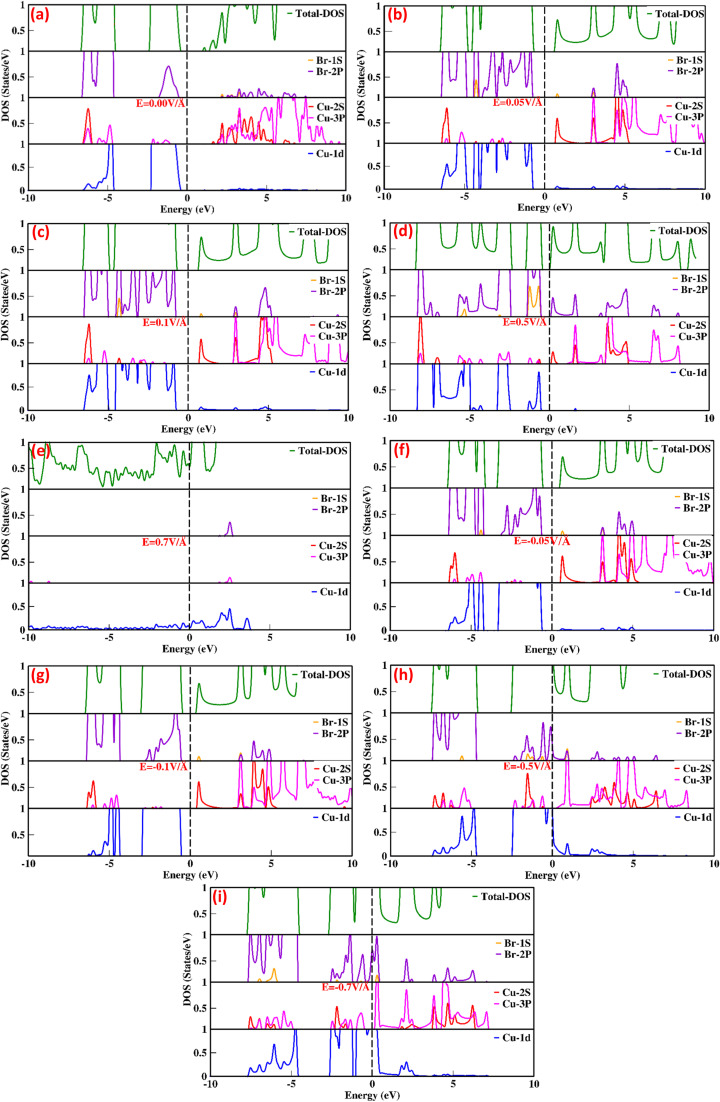
(a–i) Electric-field-induced modulation in the total density of states (TDOS) and partial density of states (PDOS) of CuBr compounds calculated using the GGA scheme.

The total density of states (TDOS) and partial density of states (PDOS) for the CuBr compound are shown in Fig. 2(a–i), providing in-depth information about the role of orbital contribution and their effect on the bandgap modulation and engineering with and without an external applied electric field (*E*). The dashed line between the valence band (VB) and conduction band (CB) represents the Fermi energy level. In the absence of an external electric field (*E* = 0 V Å^−1^), the valence band is dominated by Cu-1d and Br-2p states, and there is less contribution from Cu-2s and Cu-3p orbitals near the Fermi energy levels (the dotted lines shown in Fig. 2a). However, in the case of the conduction band, the Cu-3p orbital contributes the maximum, while Cu-2s and Br-2p contribute less. There is a very insignificant contribution from Cu-1d and Br-1s orbitals. These results confirm that Cu-d and Br-p states mostly dominated the valence band, whereas in the conduction band (CB), the s and d states of CuBr are rarely involved. The direct bandgap starts to widen with the utilization of an external applied electric field, and upon the application of 0.05 V Å^−1^ and 0.1 V Å^−1^, the bandgap increases from 0.58 to 1.58 and 1.63. The increase in band gap at 0.05 V Å^−1^ and 0.1 V Å^−1^ may be caused mainly by the interactions and orientation of electron orbital states; the Cu-1d and Br-2p states have major contributions, Cu-2s and Cu-3p states have less contribution, and Br-1s has an insignificant contribution in the valence band. However, in conduction bands, Cu-3p, Cu-2s and Br-2p orbitals have the main contribution and Cu-1d and Br-1s have less contribution. The increase in bandgap upon application of electric field (*E*) as external stimuli at 0.05 V Å^−1^ and 0.1 V Å^−1^ implies that more electron excitation energies are required from the valence to the conduction band; henceforth, the light of a higher frequency and shorter wavelength would be absorbed. The external electric field (0.1 V Å^−1^ and 0.5 V Å^−1^) causes electron energy excitation to the electrons present at lower energy levels with the main contribution of Cu-3p states at *E* = 0.0 V Å^−1^ in conduction band shift to the Cu-3p, Cu-2s and small contribution in the same energy range from Br-2p. This indicates that the Cu-2s (main contribution) and Br-2p interplay a major role, causing an increase in bandgap depending on the contribution of electrons of the same or different spin states and crystal field causing repulsion or attraction may lead to widening bandgap upon the external electric field. However, upon a further increase in the applied electric field (*E*) to 0.5 V Å^−1^, we observed that all the energy bands started to expand, and the bandgap exhibited a sharp decrease from 1.63 eV to 0.78 eV, depicting the start of the transition from the semiconductor to metallic behavior.

This decrease in bandgap generated by orbital orientations and interactions is confirmed; Fig. 2(a–i) shows that the valence band is formed because of the distinct hybridization of Cu-1d and Br-2p orbitals with less contribution from Cu-2s, Cu-3p, and Br-1s orbitals. However, in the conduction bands, Cu-3p, Cu-2s and Br-2p orbitals have the main contribution and Cu-1d and Br-1s have less contribution. In all these processes of electric field modulation, tuning the bandgap of CuBr remains direct at the *Γ*–*Γ* symmetry point of the Brillouin zone. However, on further increase in the electric field to 0.7 V Å^−1^, −0.5 V Å^−1^ the valence band crosses the Fermi energy level, vanishing the bandgap, and CuBr turned into metallic from a semiconductor. Interestingly, the applied electric field initially causes an increase in bandgap from 0.58 eV to 1.63, decreases the direct bandgap at the *Γ*–*Γ* symmetry point of the Brillouin zone from 1.63 eV to 0.78 and then overlaps conduction and valence band at *E* = 0.7 V Å^−1^ and −0.5 V Å^−1^.

**Fig. 3:**
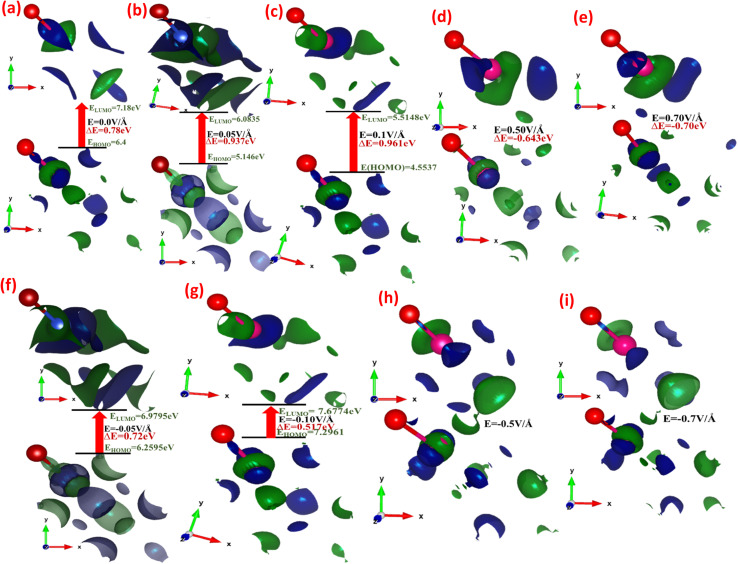
(a–i) Electric-field-induced modulation engineering and modulation of the highest occupied molecular orbitals (HOMO) or bonding orbitals and lowest unoccupied molecular orbitals (LUMO) or anti-bonding orbital energy wave functions calculated using the GGA scheme.

Fig. 3(a–i) depicts the effect of the applied electric field (*E*) on the engineering and modulation of the highest occupied molecular orbitals (HOMO) or bonding orbitals and lowest unoccupied molecular orbitals (LUMO) or anti-bonding orbital energy wave functions.

In CuBr compounds, the HOMO orbitals help Cu and Br to form CuBr bonds, which are naturally lower in energy than the LUMO orbitals that cleave the bonds between Cu and Br. To achieve lower energy stable states for CuBr, the Cu electrons interact with Br electrons to form CuBr bonding. The energy difference between HOMO and LUMO states demonstrates the ability of electrons to jump from occupied to unoccupied orbitals, demonstrating the ability of the molecular orbital to participate in chemical reactions. The energy gap (Δ*E*) between the HOMO and LUMO orbitals (which is the difference between the energies of HOMO and LUMO states (*E*_LUMO_–*E*_LOMO_)) represents the chemical activity, and a shorter gap corresponds to stronger chemical activity, as illustrated in Fig. 3(a–i), and their values are listed in Table 1. In the absence of an applied electric field (*E*) at 0.00 V Å^−1^, the energy gap (Δ*E*) between the HOMO and LUMO states of CuBr is 0.78 eV. However, there is a tendency of a shift in increase and decrease in energy gap *via* applied electric field (*E*), which may demonstrate the controlled tailored ability of chemical reactivity and electronic stability of CuBr. It is obvious from Fig. 3(a–i) that upon the increase in the electric field from 0.0 V Å^−1^ → 0.05 V Å^−1^ → 0.1 V Å^−1^, there is an increase in the energy gap (Δ*E*) from 0.78 eV to 0.961 eV, indicating more electronic stability and less chemical reactivity. However, the converse is the case upon a further increase in the electric field to 0.5 V Å^−1^, in which the HOMO and LUMO energy gaps shrink to −0.643 eV, demonstrating that electronic instability and high chemical reactivity confirm a major shift in response, which agrees well with the modulation of bandgap and PDOS calculations. The values of LUMO, HOMO energies and the energy gap between LUMO and HUMO energy levels are listed in Table 1. We observed that the overall shapes of the HOMO and LUMO energy states also changed (Fig. 3(a–i)). This is because the electric field (*E*) causes a shift in the interaction of various orbitals, such as Cu-1d, Cu-2s, Cu-3p, Br-1s and Br-2p.

**Fig. 4:**
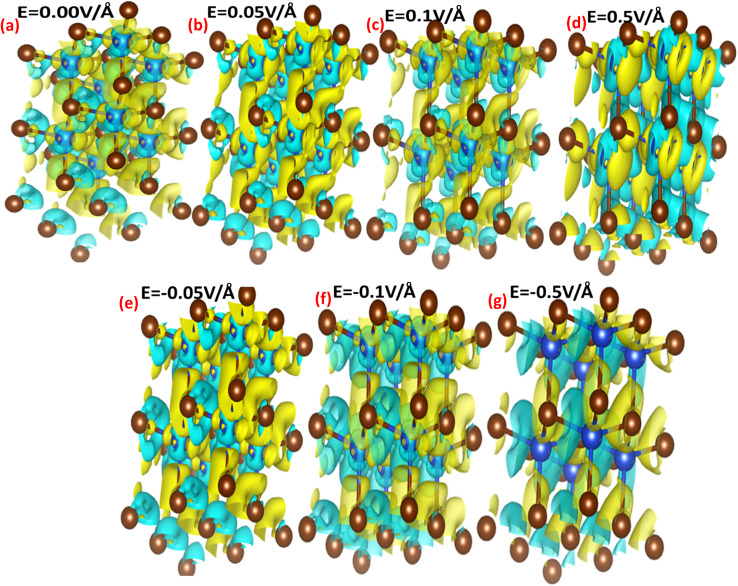
(a–g) Electric field-induced engineering and modulation of the charge density distribution in the 3D extended 2 × 2 × 2 CuBr boundary unit cells calculated using the GGA scheme.

**Fig. 5:**
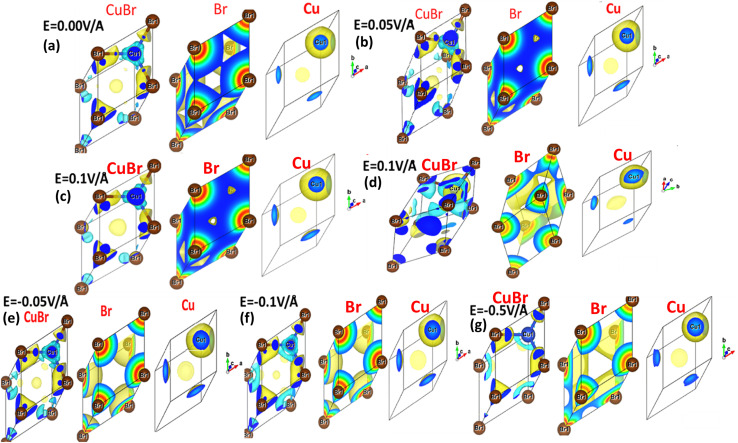
(a–g) Electric-field-induced engineering and modulation of the charge density distribution in primitive unit cells of the CuBr calculated using the GGA scheme.

The interaction between metallic Cu and halide Br in CuBr with and without an applied electric field (*E*) causing the change in charge transfer and hybridization is explored by electronic charge density distribution calculations, which is widely accepted for predicting charge density. Fig. 4(a–g) and 5(a–g) illustrate the electric field modulation of charge density distribution in CuBr in 3D 2 × 2 × 2 extended boundary unit cells and primitive unit cells.

We evaluated several patterns under various applied electric fields (0.00 V Å^−1^, 0.05 V Å^−1^, 0.1 V Å^−1^, 0.5 V Å^−1^, −0.05 V Å^−1^, −0.1 V Å^−1^, and −0.5 V Å^−1^) of the calculated charge density to validate the electronic modulation and tunability. It is evident that charges accumulate and share between the Cu metal and halide Br atoms, indicating the existence of directional shared bonding upon the application of an electric field (*E*). The sharing of mutual cations and anions shows that covalent and charge transfer reveal an ionic bonding nature. It is clear from Fig. 4(a–g) and 5(a–g) that below the applied critical external field (0.7 V Å^−1^ and −0.5 V Å^−1^), CuBr displays charge sharing and covalent bonding among the anion–anion (Br–Br) and anion–cation (Cu–Br) atoms.

However, the charge density distribution changes upon the application of a higher electric field (0.5 V Å^−1^ and −0.5 V Å^−1^), validating the shift in the nature of CuBr from semiconducting to metallic. In the absence of a field at 0.0 V Å^−1^, the charge distribution is more around the Br atoms whereas less around the Cu atoms. We observed an evident shift in charge distribution upon the field; for example, at 0.05 V Å^−1^, the charge density starts to transfer from Br to Cu atoms. The charge distribution somehow demonstrates a shift in behavior from semiconductor to metallic at higher fields of 0.5 V Å^−1^ and −0.5 V Å^−1^.

**Fig. 6:**
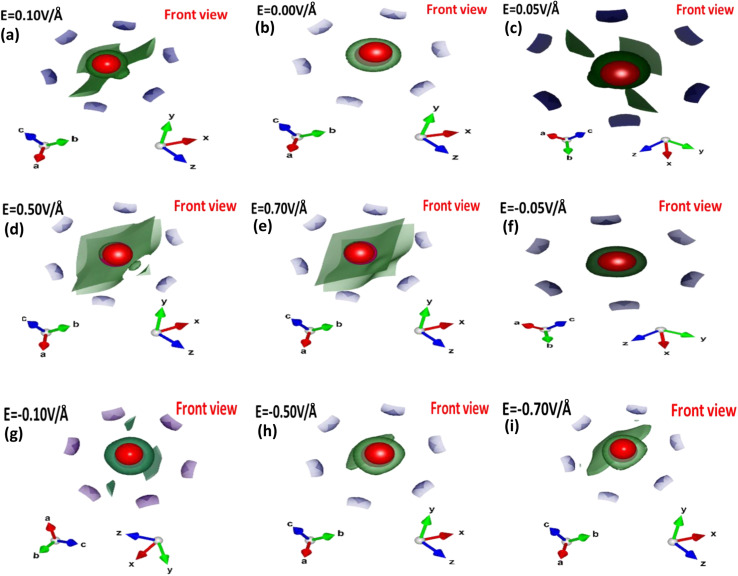
(a–i) Electric field-induced engineering and modulation of the electron localization function (ELF) in the primitive unit cell of the CuBr calculated using the GGA scheme.

To understand the role of the electric field in the bonding patterns, we performed electron localization function (ELF) calculations, which provide insight into (local) the distribution of electrons. Fig. 6(a–i) depicts the front view of the computed ELF with and without an applied electric field.

The colored regions around the Cu (red) and Br (purple) atoms represent the range (high or less) of electron localized density. In the absence of an applied electric field (0/0 V Å^−1^), high charge density is observed in neighboring Br atoms, which may be attributed to the presence of strong *σ* bonding. However, the applied electric field (*E*) results in charge transfer or accumulation from Br atoms onto Cu atoms. We observed that Cu atoms are responsible for the low charge density although the electric field causes modulation, accumulation and transfer, in which the low charge density may be due to the weakening of the *σ* bonding between the Cu atoms and the neighboring Br atoms.

**Fig. 7:**
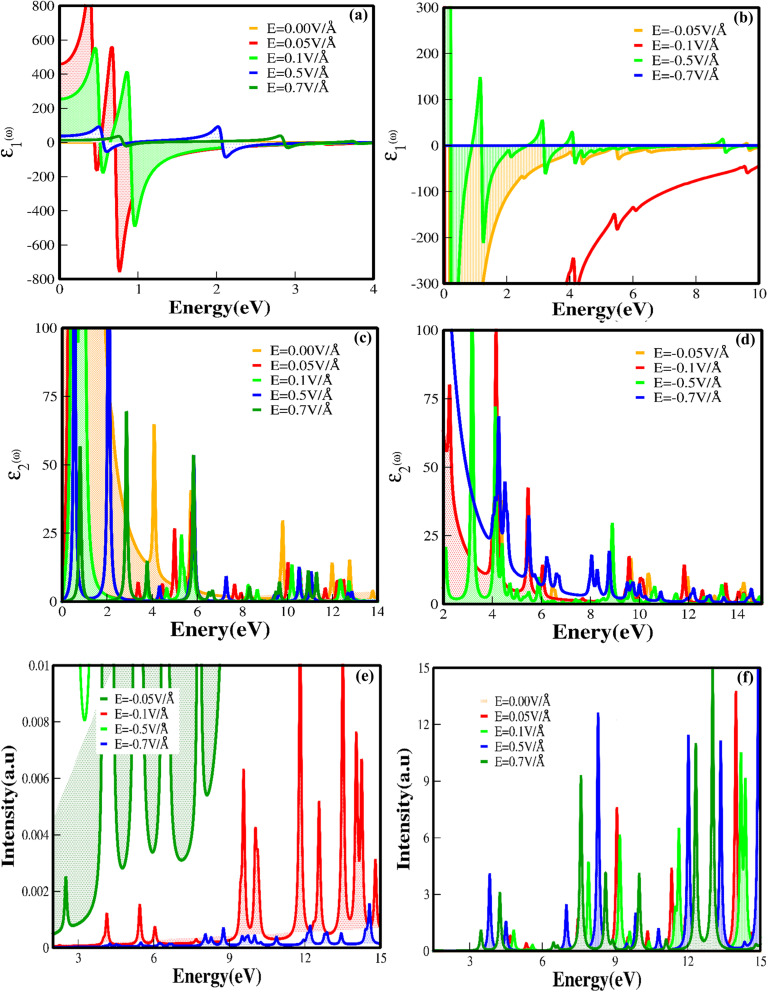
Electric-field-induced engineering and modulation of the (a and b) real part of dielectric *ε*_1_(ω), (c and d) imaginary *ε*_2_(ω) part of dielectric (e and f) electron energy loss (ELS) function of CuBr compounds calculated by GGA process.

We have investigated the optical properties of CuBr, including the optical reflectivity *R*(*ω*), refractive index (*n*(*ω*)), extinction coefficient *k*(*ω*), real *ε*_1_(*ω*) and imaginary *ε*_2_(*ω*) parts of dielectric and dielectric constants *ε*(*ω*) for the various energy ranges under the applied electric field using GGA techniques. Fig. 7(a–d) shows the real *ε*_1_(*ω*) and imaginary parts of the dielectric constant *ε*_2_(*ω*) for CuBr with respect to the applied external electric field above and below the critical external electric field (*E*_C_) as a function of energy (eV). The dielectric constant *ε*(*ω*) is a dimensionless number that describes the effect of the electric field on a material; the higher the dielectric constant, the greater the material's tendency to reduce any field set up inside it. The calculated values of dielectric constant *ε*(*ω*) with and without an applied electric field (*E*) revealed that CuBr displays a dielectric response. Based on GGA approximations, the values of the real part of dielectric *ε*_1_(*ω*) as a function of the applied electric field (*E*) are in the range of 5.57 at 0.5 V Å^−1^ to 150 at −0.3 V Å^−1^, and the values of the imaginary part of dielectric *ε*_2_(*ω*) are in the range of −324 to 149 at −0, = 0.3 V Å^−1^ and −0.5 V Å^−1^. The real part of dielectric function *ε*_1_(*ω*) of CuBr reveals that the band gap is inversely proportional to the value of dielectric constant *ε*(*ω*), implying that the band gap increases as the dielectric constant decreases, which may be explained based on the Penn model.^42,43^ The detailed individual (at different peaks) and average values of the real part of dielectric function *ε*_1_(*ω*), the imaginary part of dielectric *ε*_2_(*ω*) and dielectric constant *ε*(*ω*) under the applied electric field (*E*) (with and without) are listed in Table 2. It is clear from Fig. 7(a and b) that in the absence of an applied electric field, the *ε*_1_(*ω*) increases as the energy increases. The magnitude of the peak of the real dielectric decreases as the applied electric field (up to 0.1 V Å^−1^ and −0.1 V Å^−1^) increases, suggesting an increase in the band gap. There is a sharp increase in *ε*_1_(*ω*) as the electric field increases from 0.1 V Å^−1^ to 0.5 V Å^−1^ because the bandgap decreases, demonstrating the behavior shift from semiconducting to conducting with the applied field.

**Table 2:** Detailed individual and average values of electric-field-induced real part of dielectric *ε*_1_(*ω*), imaginary part of dielectric *ε*_2_(*ω*) and dielectric constant *ε*(*ω*) of CuBr

S. no	Electric field (V Å^−1^)	1^st^ peak		2^nd^ peak		3^rd^ peak		4^th^ peak		5^th^ peak		Avg imaginary	Average real	Dielectric const
		*ε*_2_(*ω*)	*ε*_1_(*ω*)	*ε*_2_(*ω*)	*ε*_1_(*ω*)	*ε*_2_(*ω*)	*ε*_1_(*ω*)	*ε*_2_(*ω*)	*ε*_1_(*ω*)	*ε*_2_(*ω*)	*ε*_1_(*ω*)	*ε*_2_(*ω*)	*ε*_1_(*ω*)	*ε*(*ω*)
1	0.00	60	−60	40	−40	30	−35	14	−34.8	15	32.9	31.8	−16.9	14.9
2	0.05	7.34	−6.60	7.03	1.50	26.49	11.40	32.98	2.70	6.72	9.90	16.11	3.78	19.89
3	0.10	6.3	−1.61	5.0	2.56	24.7	14	35.2	−4.0	—	—	17.8	2.73	20.53
4	0.50	5.57	3.63	53.4	27	9.3	5.0	2.4	3.0	5.3	3.2	15.19	8.366	23.55
5	0.70	68	39	14.1	6.5	53.9	28.3	3.6	−1.5	3.2	−1.7	28.56	14.12	42.68
6	−0.05	11.35	−13.71	14.4	−4.62	37.92	−3.14	27.73	−7.40	7.95	−5.50	19.78	−6.79	12.99
7	−0.10	79.8	−148.1	100	−134	42.53	−43.6	13.95	−43.0	2.76	−27.1	47.80	−79.16	31.36
8	−0.50	20.84	149.4	118	−8.90	72.28	55.21	21.96	29.09	7.42	−11.2	39.79	42.70	82.49

Fig. 7(c and d) illustrates the imaginary part of the dielectric constant *ε*_2_(*ω*)as a function of the given photon energy (eV) above and below the critical applied electric field (0.0 V Å^−1^, 0.05 V Å^−1^, 0.1 V Å^−1^, 0.5 V Å^−1^, 0.7 V Å^−1^) with respect to the reverse field (−0.05 V Å^−1^, −0.1 V Å^−1^, −0.5 V Å^−1^, −0.7 V Å^−1^) for the CuBr system. To explore excitons, we must consider the imaginary part of dielectric *ε*_2_(*ω*) because it contains a signature of exciton energies. Imaginary dielectrics *ε*_2_(*ω*) represent four major peaks in the energy ranges 3.8–4.1 eV, 4–4.4 eV, 5.7–6.0 eV, and 9.5–10 eV for the CuBr system at 0.0 V Å^−1^, corresponding to the four absorptions. The imaginary dielectric absorption peaks decrease as the applied external field increases and reach a minimum value above the critical field (0.5 V Å^−1^). It is noteworthy that the absorption peak shifts from lower energy to higher energy with the utilization of an external applied field. Furthermore, the sharpness of the absorption peak increases as the electric field increases. Few absorption peaks exhibit an increase in width, which could be due to the mixed transition. The major contributions to the optical transition at low field (0.0 V Å^−1^, 0.05 V Å^−1^, −0.05 V Å^−1^, 0.1 V Å^−1^ and −0.1 V Å^−1^) belong to the Cu-d and Br-p states. However, at the higher field (0.5 V Å^−1^, −0.5 V Å^−1^, 0.7 V Å^−1^ and −0.7 V Å^−1^), the major contribution shifts to the s–p–d states.

The shift of absorption edge towards the higher energies with applied external electric field (at 0.5 V Å^−1^, 0.7 V Å^−1^ and −0.5 V Å^−1^ and above) illustrates a reduction in bandgap, as shown in Fig. 7(a–d). The optical absorption edge occurs at the *Γ* point of the Brillouin zone (BZ) between the conduction and valence bands, demonstrating a threshold for direct optical transition. The interesting fact to notice here is that *ε*_2_(*ω*) decreases as the positive applied electric field increases and even modulates over the negative applied electric field. This is because with an applied electric field, CuBr becomes polarized, but the field produced due to the polarization of the CuBr minimizes the effect of the external field. These results indicate that the dielectric response of CuBr with an applied electric field (*E*) confirms its potential usage in controlled optoelectronics. Maximum energy loss functions above and below the critical field with the negative field are demonstrated in Fig. 7(e and f), which are confined to the energy regions where the electrons are not typically restricted to their lattice site and execute oscillation upon light exposure. It is obvious that the maximum energy loss function (ELS) minimum value refers to the higher value of the imaginary part of the dielectric, depending on the energy band gap, which varies with the applied external electric field. For example, the maximum energy loss function increases as the band gap decreases with the application of a higher field, resulting in a transition from semiconducting to a metallic behavior.

The complex refractive index (*ñ*) is a crucial optical property of material given by the following formula:^44,45^
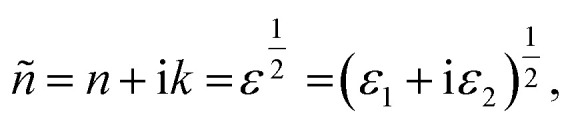
where *n* represents the real refractive index and *k* represents the attenuation index or extinction coefficient. We evaluate both *n* and *k* for CuBr by utilizing the imaginary part of the dielectric function using the following equation:^44,45^
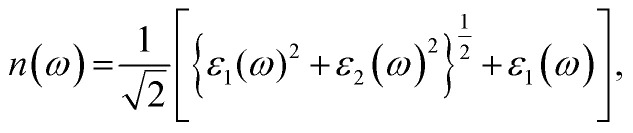

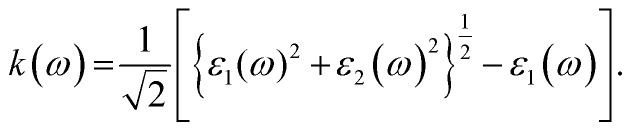


**Fig. 8:**
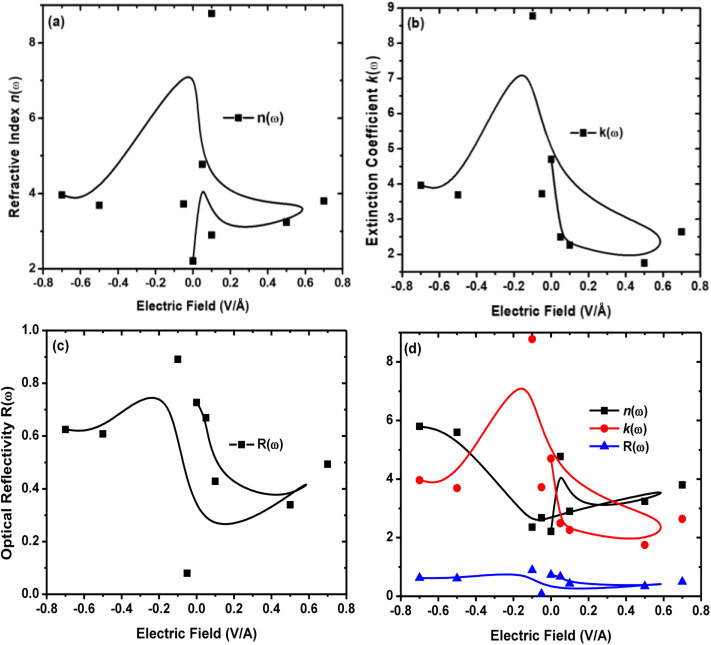
Electric-field-induced engineering and modulation of the (a) refractive index *n*(*ω*), (b) extinction coefficient *k*(*ω*), (c) optical reflectivity *R*(*ω*) and (d) comparison between refractive index *n*(*ω*), extinction coefficient *k*(*ω*) and optical reflectivity *R*(*ω*) of CuBr compounds calculated by applying the GGA process.

The calculated values of the refractive index *n*(*ω*) and extinction coefficient *k*(*ω*) as a function of the given photon energy (eV) above and below the critical applied electric field (0.00 V Å^−1^, 0.05 V Å^−1^, 0.1 V Å^−1^, 0.5 V Å^−1^, 0.7 V Å^−1^) and with respect to the reverse field (−0.05 V Å^−1^, −0.1 V Å^−1^, −0.5 V Å^−1^, −0.7 V Å^−1^) for the CuBr system are shown in Fig. 8(a–d) and listed in Table 3.

**Table 3:** Detailed individual and average values of electric-field-induced the refractive index *n*(*ω*), extinction coefficient *k*(*ω*) and optical reflectivity *R*(*ω*) of CuBr

S. no.	*E* (V Å^−1^)	*k*_1st_(*ω*)	*k*_2nd_(*ω*)	*k*_3rd_(*ω*)	*k*_4th_(*ω*)	*k*_5th_(*ω*)	*k*_avg_(*ω*)	*n*_avg_(*ω*)	*R*_avg_(*ω*)
1	0.00	3.52	6.94	6.36	5.90	1.27	4.70	2.21	0.727
2	0.05	2.86	1.72	2.95	3.89	1.01	2.492	4.77	0.669
3	0.10	2.01	1.23	2.68	3.13	—	2.26	2.9	0.428
4	0.50	1.22	4.05	1.66	0.648	1.22	1.75	3.24	0.339
5	0.70	4.43	2.12	4.03	1.64	0.98	2.64	3.8	0.493
6	−0.05	3.96	3.14	4.53	4.24	2.75	3.72	2.686	0.08
7	−0.10	12.57	12.27	7.22	6.67	5.21	8.782	2.36	0.891
8	−0.50	0.850	7.97	4.22	1.917	3.50	3.691	5.6	0.608
9	−0.70	5.87	4.73	3.94	2.92	2.35	3.96	5.8	0.625

The refractive index *n*(*ω*) increases as the band gap decreases upon the utilization of the applied external electric field, reaching the maximum value at the critical field (3.8 at 0.7 V Å^−1^ and 5.8 at −0.7 V Å^−1^) and following the trend of the real part of dielectric *ε*_1_(*ω*) that confirms semiconducting to metallic band transition. Fig. 8(c) demonstrates that the optical reflectivity spectra with respect to the applied electric field at given phonon energies provide basic information about various critical points of transition. We extracted the electric field tailored, manipulated and controlled optical reflectivity for CuBr below, above and in a negative polarization applied electric field using the fundamental relation:^46^
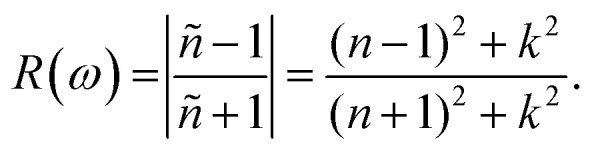


The complex refractive index *n*(*ω*) demonstrates an optical response to electromagnetic waves or light in two major parts: the refractive index, *n*(*ω*), and extinction coefficient, *k*(*ω*). These are energy and frequency dependent parameters, respectively.

The optical reflectivity *R*(*ω*) with and without an applied electric field for the CuBr system is calculated and represented in Fig. 8(c). We observed that optical reflectivity decreases and shifts toward lower values as the applied electric field increases. Such a shift and decrease in optical reflectivity *R*(*ω*) with a higher applied field (*E*) confirm the transformation from semiconducting CuBr to metallic. The maximum optical reflectivity *R*(*ω*) values at zero photon energy (0 eV) are associated with the highest absorption energy.

## Conclusions

In summary, we first successfully reported the effect of the external applied electric field (*E*) on electronic bandgap engineering and modulation, causing changes and shifts in structural, electronic and optoelectronic properties of CuBr *via* plane-wave basis set and pseudopotentials (pp) using the generalized gradient approximation (GGA) based on density functional theory (DFT). We observed that when the external electric field was applied, there was a significant increase in the bandgap, such as from 0.58 at 0.0 V Å^−1^ to 1.63 at 0.1 V Å^−1^ (about 280% increase). This modulation in the electronic bandgap *via* the applied electric field (*E*) results in a behavioral shift from semiconducting to metallic. The partial density of states (PDOS), charge density and electron localization function (ELF) calculations reveal that the applied electric field (*E*) modulates the orbital contribution and leads to the main contribution of Cu-1d, Br-2p, Cu-2s, Cu-3p, and Br-1s orbitals in the valence band and Cu-3p, Cu-2s and Br-2p, Cu-1d and Br-1s orbitals in the conduction band, significantly confirming the controlled optoelectronic properties. Additionally, we found that the chemical reactivity and electronic stability of CuBr may be controlled by tuning and tailoring shifts in HOMO and LUMO states with an increase or decrease in gap *via* an applied electric field (*E*). The increase in the electric field from 0.0 V Å^−1^ → 0.05 V Å^−1^ → 0.1 V Å^−1^ causes the increase in the energy gap to lead to electronic stability and less chemical reactivity. However, the converse is the case upon a further increase in the electric field to 0.5 V Å^−1^, where the gap shrinks to 0.78, leading to electronic instability and high chemical reactivity that indicate a major shift in response. This is further confirmed by observing modulation in the bandgap and PDOS calculations. Optical properties, such as optical reflectivity *R*(*ω*), refractive index (*n*(*ω*)), extinction coefficient *k*(*ω*), real *ε*_1_(*ω*) and imaginary *ε*_2_(*ω*) parts of dielectric and dielectric constant *ε*_1_(*ω*) for various energies ranging in eV under the applied electric field, confirm the controlled optoelectronic response in CuBr. This work offers valuable insights and an in-depth study of the fascinating photophysical properties of CuBr films *via* an applied electric field, and it will open a prospect to their utilization in various applications.

## Conflicts of interest

I hereby confirm that the work reported in this manuscript is novel and has no conflict of interest.

## Data availability

The raw/processed data required to reproduce these findings cannot be shared at this time as the data also forms part of an ongoing study. Furthermore, the data may be provided on request.

## Supplementary Material
